# Proteome of *Staphylococcus aureus* Biofilm Changes Significantly with Aging

**DOI:** 10.3390/ijms23126415

**Published:** 2022-06-08

**Authors:** Md. Arifur Rahman, Ardeshir Amirkhani, Durdana Chowdhury, Maria Mempin, Mark P. Molloy, Anand Kumar Deva, Karen Vickery, Honghua Hu

**Affiliations:** 1 Surgical Infection Research Group, Faculty of Medicine, Health and Human Sciences, Macquarie University, Sydney 2109, Australia; durdanachowdhury@gmail.com (D.C.); maria.mempin@mq.edu.au (M.M.); anand.deva@mq.edu.au (A.K.D.); karen.vickery@mq.edu.au (K.V.); 2 Australian Proteome Analysis Facility, Macquarie University, Sydney 2109, Australia; ardeshir.amirkhani@mq.edu.au (A.A.); mark.molloy@mq.edu.au (M.P.M.)

**Keywords:** *Staphylococcus aureus*, biofilms, proteome, TMT, mass spectrometry, aging, metabolic processes, biosynthetic processes, transport systems, stress responses

## Abstract

*Staphylococcus aureus* is a notorious biofilm-producing pathogen that is frequently isolated from implantable medical device infections. As biofilm ages, it becomes more tolerant to antimicrobial treatment leading to treatment failure and necessitating the costly removal of infected devices. In this study, we performed in-solution digestion followed by TMT-based high-throughput mass spectrometry and investigated what changes occur in the proteome of *S. aureus* biofilm grown for 3-days and 12-days in comparison with 24 h planktonic. It showed that proteins associated with biosynthetic processes, ABC transporter pathway, virulence proteins, and shikimate kinase pathway were significantly upregulated in a 3-day biofilm, while proteins associated with sugar transporter, degradation, and stress response were downregulated. Interestingly, in a 3-day biofilm, we observed numerous proteins involved in the central metabolism pathways which could lead to biofilm growth under diverse environments by providing an alternative metabolic route to utilize energy. In 12-day biofilms, proteins associated with peptidoglycan biosynthesis, sugar transporters, and stress responses were upregulated, whereas proteins associated with ABC transporters, DNA replication, and adhesion proteins were downregulated. Gene Ontology analysis revealed that more proteins are involved in metabolic processes in 3dwb compared with 12dwb. Furthermore, we observed significant variations in the formation of biofilms resulting from changes in the level of metabolic activity in the different growth modes of biofilms that could be a significant factor in *S. aureus* biofilm maturation and persistence. Collectively, potential marker proteins were identified and further characterized to understand their exact role in *S. aureus* biofilm development, which may shed light on possible new therapeutic regimes in the treatment of biofilm-related implant-associated infections.

## 1. Introduction

It is projected that about 65 to 85% of all bacterial infections are associated with biofilms [1,2]. *Staphylococcus aureus* is a notorious biofilm-producing pathogen and together with coagulase-negative staphylococci, is frequently isolated from biofilm infections associated with implantable medical devices such as catheters, prosthetic joints, breast implants, and pacemakers [3,4,5]. Once developed, biofilm is enormously hard to eliminate due to its high tolerance to both antimicrobials and host immune defenses [6]. Eradication often requires painful and expensive removal or replacement of contaminated medical devices.

Biofilm is a multilayered structure comprising bacterial communities embedded in a self-produced matrix containing extracellular polymeric substances (EPS). In *Staphylococcus* biofilms, proteins such as fibronectin-binding proteins (FnBPs) and biofilm-associated proteins (Bap), polysaccharide intercellular adhesion (PIA), extracellular DNA (eDNA), and teichoic acids are components of EPS matrix [7,8,9,10]. PIA is thought to be a principal extracellular component (80–85%) and is mainly composed of β-1,6-linked N-acetylglucosamine residues. In vitro, PIA is generated from UDP-N-acetylglucosamine through intercellular adhesion (*ica*) locus products [11]. In some strains of *S. aureus*, biofilm development is dependent upon the *ica* locus [*icaR* (regulatory) and *icaADBC* (biosynthetic) genes] [10,12]; however, *ica*-independent mechanisms are sufficient for biofilm formation in some strains of *S. aureus* and coagulase-negative staphylococci [10,13]. Studies by Houston et al. (2011) demonstrated that fibronectin-binding proteins (FnBPs) can mediate *S. aureus* biofilm development via a vital role by the major autolysin (*Atl*), *agr*, and *sigB* regulation [14]. However, accumulation-associated protein (*Aap*) plays a significant role in PIA-independent biofilm formation in *Staphylococcus epidermidis* [15,16].

Biofilm-associated protein (Bap) plays a significant function among the adhesive proteins involved in the formation of biofilms, which is crucial for both initial attachment and intercellular accumulation during the growth of *S. aureus* biofilm. The Bap gene is also found in many coagulase-negative staphylococci and several other staphylococcal species [17]. In addition, the differential expression and synthesis of these molecular variables during the distinct phases of biofilm development are tightly controlled by various biofilm regulators as listed by Graf et al. (2019) such as *SarA*, *AgrA*, *RNAIII*, *Rot*, *IcaR*, *CodY*, *Spx*, and others [18].

Global advances can be especially advantageous in unraveling the complex interaction among these regulatory networks and in identifying proteins with vital functions in biofilm development. However, most of the omics studies focus on the profiling of early-stage biofilm development and/or up to 3-days [19,20,21] but the physiology of older biofilm is likely to be different. Certainly, older biofilm is more tolerant to disinfectants [22].

In the current study, we aimed to construct a comprehensive quantitative proteomic framework to bridge the knowledge gap in *S. aureus* cells as they progress through planktonic, mature and prolonged biofilms.

## 2. Results

### 2.1. TMT Identification of Differentially Regulated Proteins

**Figure 1:**
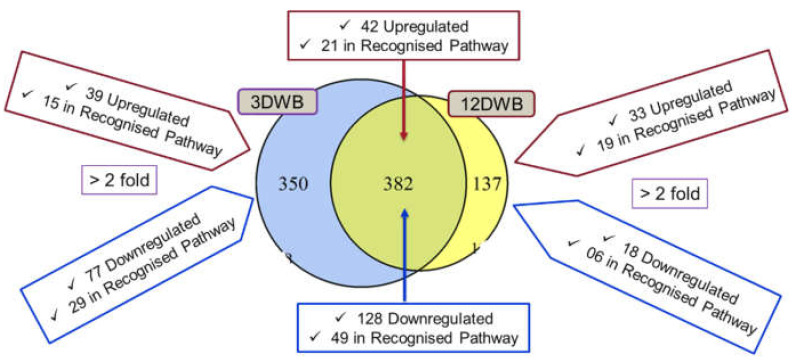
Venn diagram shows the dispersion of common and significant differential proteins in 3dwb and 12dwb. Pathway analysis was performed using KEGG.

A total of 1636 non-redundant proteins with at least one unique peptide and <1% FDR were identified and quantitated. When we processed our database through the TMTPrePro R package, we considered the cutoff range > 1.5-fold, *p* < 0.05. Among them, 350 and 137 significant differentially regulated proteins and 382 common proteins were identified compared to planktonic bacteria in 3dwb and 12dwb, respectively (Figure 1). In this study, however, we specifically focused only on proteins dysregulated > 2-fold, *p* < 0.05, to make the changes more biologically relevant. Of these, 39 and 33 proteins were significantly (>2-fold) upregulated compared to planktonic bacteria in 3dwb and 12dwb, respectively (Figure 1).

Of these, 15 and 19 proteins were detected in recognised protein pathways in 3dwb and 12dwb, respectively (Figure 1). In contrast, 77 and 18 proteins were significantly (> 2-fold) downregulated in 3dwb and 12dwb, respectively. Of these, 29 and 6 proteins were detected in recognised protein pathways in 3dwb and 12dwb, respectively (Figure 1). Among the common proteins between 3dwb and 12wb, 42 proteins were significantly upregulated (>2-fold), of which 21 proteins were involved in recognised pathways, and 128 proteins were significantly downregulated (>2-fold), of which 49 proteins were involved in recognised pathways (Figure 1).

### 2.2. GO Analysis and Annotation of Differentially Regulated Proteins

**Figure 2:**
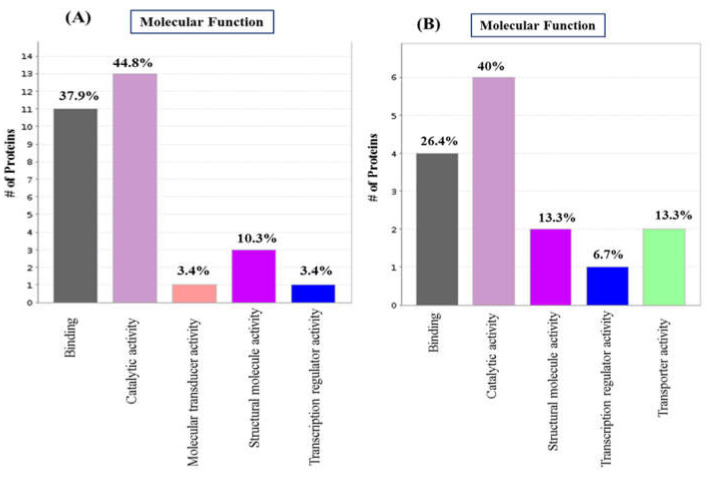
Classification of the unique/exclusive differentially regulated proteins of 3dwb and 12dwb based on their functional annotations using Gene Ontology. (**A**) GO Molecular Function for 3dwb; (**B**) GO Molecular Function for 12dwb.

We performed GO functional annotation for unique/significant differentially regulated proteins of 3dwb and 12dwb. In this analysis we considered only proteins dysregulated > 2-fold and *p* < 0.05. PANTHER analysis revealed the 5 most represented molecular functions in the complete global protein repository for both 3dwb and 12dwb (Figure 2). Among them, proteins involved in catalytic activity (44.8%) were the highest, followed by binding (37.9%) and structural molecule activity (10.3%) in 3dwb. In 12dwb, proteins involved in catalytic activity (40%) were the highest, followed by binding (26.4%), and structural molecule activity and transporter activity (13.3%).

**Figure 3:**
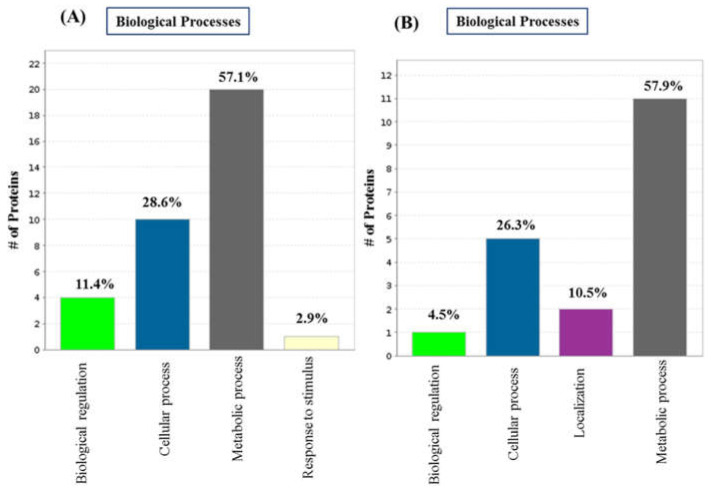
Classification of the unique/exclusive differentially regulated proteins of 3dwb and 12dwb based on their functional annotations using Gene Ontology. (**A**) GO Biological Processes for 3dwb; (**B**) GO Biological Processes for 12dwb.

Biological process evaluation identified the four most represented biological processes (Figure 3) for both 3dwb and 12dwb. Of the proteins significantly upregulated/downregulated > 2-fold, 20 proteins (57.1%) were involved in metabolic processes, 10 proteins identified (28.6%) in cellular processes, and 4 proteins (11.4%) were involved in biological regulations in 3dwb. In the 12-day biofilm, a similar percentage of proteins involved metabolic processes. 11 proteins (57.9%) and 5 proteins (26.3%) were involved in cellular processes; in contrast, two proteins (10.5%) were involved in localisations. These results suggest that more proteins are involved in metabolic processes in 3dwb compared with 12dwb.

### 2.3. Significantly Upregulated Proteins and Pathway Analysis in 3dwb

**Figure 4:**
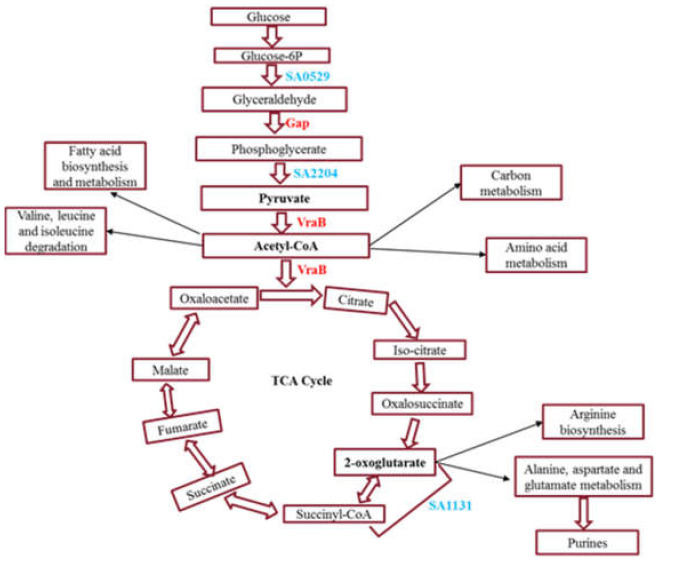
Exclusive differentially regulated proteins involved in central metabolism in 3dwb. Red names denote encoded genes with increased abundance while blue names denote encoded genes with decreased expression.

The proteins that are significantly upregulated in 3-day biofilm in comparison with planktonic and 12-day biofilm are listed in Table S1. Of these proteins, acetyl-CoA acetyltransferase (> 3.5-fold upregulated), a cytoplasmic enzyme, was uniquely identified in 3-day biofilm and is involved in multiple metabolic pathways including fatty acid and pyruvate metabolism, indicating that this enzyme plays a central role in energy metabolism (Figure 4), regulation of protein function under nutrient and oxygen-limiting conditions, and thus ensures the survivability of biofilm [23,24,25,26].

Further, glyceraldehyde-3-phosphate dehydrogenase (*gap*) is uniquely/significantly upregulated in a 3-day biofilm, is involved in glycolysis pathway, and is responsible for energy metabolism, which is also part of the central metabolism (Figure 4).

Cystathionine gamma-synthase (*metB*), 3-phosphoshikimate 1-carboxyvinyltransferase (*aroA*), and histidinol-phosphate aminotransferase were significantly upregulated in 3dwb and involved in multiple biosynthetic processes including amino acids, indicating an increase in the synthesis of amino acids. Selective amino acid uptake by the biofilm is significant for energy utilization for maintenance of a proper redox balance, adaptation to diverse environmental conditions, and nutrient availability thereby ensuring survivability [27].

We identified that N-acetylmannosamine-6-phosphate 2-epimerase (*nanE*) and UDP-N-acetylglucosamine 1-carboxyvinyltransferase (*murA*) were significantly upregulated. These proteins are involved in amino sugar and nucleotide sugar metabolism and peptidoglycan biosynthesis. They are linked with the synthesis of cell-wall components which may lead to EPS matrix deposition in the 3-day biofilm. Other upregulated cytoplasmic enzyme 2-amino-4-hydroxy-6-hydroxymethyldihydropteridine pyrophosphokinase (*folk*) is a part of folate biosynthesis which may favour intracellular accumulation of folate. 30S ribosomal protein S14 (*rpsN*) and 30S ribosomal protein S5 (*rpsE*) are significantly upregulated, and such proteins may help cells adapt to the diverse conditions and guarantee cellular metabolism. The function of other upregulated proteins is unknown (Table S1).

### 2.4. Significantly Upregulated Proteins and Pathway Analysis in 12dwb

The proteins that are significantly upregulated in 12dwb in comparison with planktonic and 3dwb are listed in Table S2. Proteins such as octanoyltransferase (*lipM*), acetoin reductase (*butA*), and nitrite reductase are involved in nitrite reduction; arginase (*arg*) and 5-(carboxyamino)imidazole ribonucleotide mutase are involved in multiple metabolic processes including galactose (*lacAE*) and pyrimidine metabolism (*pyrEGHR*). A cytoplasmic enzyme 2-oxoisovalerate dehydrogenase is part of biosynthesis of antibiotics, biosynthesis of secondary metabolites, valine, leucine and isoleucine degradation, and propanoate metabolism. Ribose-phosphate pyrophosphokinase (*prs*) is a cytoplasmic enzyme involved in multiple metabolisms-associated pathways including pentose phosphate and the glycolytic pathway. The high abundance of ribose may lead to metabolic change associated with TCA cycle stress and biofilm formation [28,29]. A cytoplasmic enzyme 4′-phosphopantetheinyl transferase belongs to pantothenate and CoA biosynthesis was also upregulated. Proteins associated with cytoplasmic membrane include cytochrome C oxidase subunit III (*qoxC*) involved in oxidative phosphorylation and preprotein translocase subunit *SecY* which is involved in quorum sensing, bacterial secretion systems, and protein export. Phosphotransferase system (PTS) lactose-specific transporter subunits IICB is a cytoplasmic-membrane protein and is involved in metabolic pathways such as nitrogen metabolism, galactose metabolism, and phosphotransferase system.

A cytoplasmic enzyme aminoacyltransferase (*femX*) significantly upregulated in 12dwb and involved in peptidoglycan biosynthesis, plays an important role in cell-wall synthesis and may be very important for biofilm maintenance. The function of other upregulated proteins is unknown (Table S2).

### 2.5. Significantly Downregulated Proteins and Pathway Analysis in 3dwb

The proteins that are significantly downregulated in 3dwb in comparison with planktonic and 12dwb are listed in Table S3. Of these, response regulator *GraR* and alanine-phosphoribitol ligase (*dltA*) are cytoplasmic enzymes involved in a two-component system and cationic antimicrobial peptide (CAMP) resistance. A cytoplasmic membrane protein PTS fructose transporter subunit IICO is a part of fructose and mannose metabolism, oligoribonuclease and 7-cyano-7-deazaguanine reductase are cytoplasmic enzymes involved in sulfur metabolism and folate biosynthesis, indicating they reduce the utilisation of these molecules in 3dwb. Tryptophanyl-tRNA synthetase (*trpS*), and methionine-tRNA ligase (*metG*) are cytoplasmic enzymes involved in aminoacyl-tRNA biosynthesis and selenocompound metabolism. We identified several stress responses associated proteins that were significantly/uniquely downregulated in 3-day biofilm such as cold-shock protein, *Clp* protease, thioredoxin reductase, glyoxalase, catalase, and general stress protein as well as other proteins (Table S3).

In addition, we observed 2-oxoglutarate ferredoxin oxidoreductase subunit alpha (*SA1131*) was significantly downregulated in 3dwb. This enzyme is involved in multiple pathways including pyruvate metabolism and tricarboxylic acid (TCA) cycle and is vital for the reductive TCA cycle which takes the opposite shape of the TCA cycle and is primarily disseminated in anaerobic autotrophs. 2-oxoglutarate ferredoxin oxidoreductase catalyses the carbon dioxide fixation reaction to succinyl-CoA, resulting in 2-oxoglutarate production (Figure 4). This reaction needs strong reducing power (occurs in a reducing environment), and ferredoxin plays an important role in maintaining this redox balance. Studies have reported that 2-oxoglutarate ferredoxin oxidoreductase is important for maintaining bacterial growth under anaerobic conditions [30], and it also plays a role as a source for low-potential electron equivalents for nitrogen fixation [31]. A study by Dorner et al. (2002) reported that 2-oxoglutarate ferredoxin oxidoreductase plays a pivotal role in the metabolism of aromatic compounds in the absence of molecular oxygen by catalysing the transfer of two electrons from decreased ferredoxin to benzoyl-CoA driven by ATP. They described that reducing the benzene ring is a cycle that is energetically and mechanistically hard and needs exceptionally low redox potential and ferredoxin functions as an electron shuttle between the TCA cycle and benzoyl-CoA reductase by combining the oxidation of the benzoyl-CoA pathway end product, acetyl-CoA, with the decrease in the aromatic ring [31]. Therefore, it can be concluded that the identified proteins involved in the central metabolism pathways maintain biofilm growth under diverse environments by providing an alternative metabolic route to utilize energy.

Other downregulated proteins include phenol soluble modulin which is cytoplasmic enzyme belonging to the quorum-sensing pathway, ribonuclease H is a cell-wall enzyme belonging to the DNA replication pathway. Proteins are uniquely/significantly downregulated in 3-day biofilms such as oligoribonuclease, ribose 5-phosphate isomerase (*rpiA*), 6-phospho 3-hexuloisomerase, glutamine synthetase (*glnA*), phosphoglyceromutase, polynucleotide phosphorylase (*pnp*), ribonucleotide-diphosphate reductase subunit beta (*nrdF*), involved in multiple metabolisms-associated pathways including pentose phosphate and RNA degradation. Additionally, the function of other downregulated proteins is unknown (Table S3).

### 2.6. Significantly Downregulated Proteins and Pathway Analysis in 12dwb

The proteins that are significantly downregulated in 12dwb in comparison with planktonic and 3dwb are listed in Table S4. Of these, DNA polymerase III subunit delta, and urease subunit gamma (*urea*), are involved in several metabolic pathways including mismatch repair, and DNA replication. The downregulated 30S ribosomal protein S14, cytoplasmic enzyme, is involved in ribosome metabolism. Proteins associated with transcription elongation factor (*GreA*), ribosome recycling factor, and cell division protein (*FtsK*) were significantly downregulated, indicating decreased metabolism of translation and post-transcriptional modification in 12dwb. Hemin ABC transporter ATP-binding protein (*hrtA*) and amino acid ABC transporter substrate-binding protein are cytoplasmic membrane enzymes belonging to the ABC transporters pathway. Fibrinogen-binding protein encoded by SA1004 significantly identified in 12-day biofilm is a virulence factor protein, and responsible for *S. aureus* infection in humans, also downregulated in another *S. aureus* proteomic study [32].

### 2.7. Differentially Regulated Proteins Common to Both 3dwb and 12dwb

A total of 170 proteins (> 2-fold, *p* < 0.005) were detected in both 3dwb and 12dwb. Of these, 42 proteins were upregulated and 21 of these proteins were in recognised pathways whereas 128 proteins were downregulated, and 49 of which are in recognised pathways. Among the upregulated proteins, amino acid biosynthesis and metabolism, purine and pyrimidine metabolism associated proteins showed an increased abundance trend in 3dwb (Table S5). However, most of the upregulated proteins showed an even distribution. On the other hand, downregulated proteins were higher in the categories of amino acid biosynthesis, energy metabolism, translation, and post-translational modification indicating a lower metabolic activity profile in 12dwb (Table S6) and supporting the overall perspective of biofilms as slow-growing, metabolically lethargic communities which are more predominant for 12-day biofilm.

### 2.8. Protein-Protein Interaction (PPI) Analysis

Because PPI plays an important role in most cellular biological processes, a PPI network was constructed using STRING online PPI prediction software (Figure S1) of the 116 and 51 significantly > 2-fold differentially expressed proteins in 3dwb and 12dwb respectively. In 3dwb, a set of significant differentially regulated proteins was found to be actively interacting including: 30S ribosomal protein S14 (*rpsN*), 30S ribosomal protein S5 (*rpsE*), proline-tRNA ligase (*proS*), thymidylate kinase (*tmk*), adenine phosphoribosyltransferase (*apt*), 5′-nucleotidase (*sasH*), ribonucleotide-diphosphate reductase subunit beta (*nrdF*), thioredoxin reductase (*trxB*), DNA-directed RNA polymerase subunit alpha (*rpoA*), 50S ribosomal protein L25 (*ctc*).

For 12dwb, there was an active interaction between preprotein translocase subunit (*secY*), 50S ribosomal protein L18 (*rplR*), 50S ribosomal protein L4 (*rplD*), orotate phosphoribosyltransferase (*pyrE*), ribose-phosphate pyrophosphokinase (*prs*), uridylate kinase (*pyrH*), urease subunit gamma (*ureA*), and hemin ABC transporter ATP-binding protein (*hrtA*). This demonstrates not only that proteins are differentially expressed in early biofilm compared to more mature biofilm but that the protein-protein interactions are different. Targeted experimental and PPI analysis will increase our understanding of how proteins are biologically connected and their functions in differently aged biofilm.

### 2.9. Validation of TMT Data with Real-Time qPCR

Individual normalized qPCR results are shown in Table S7. Upregulated and downregulated protein expression and gene expression results were expressed in fold change. The qPCR results showed that, in 12dwb, *prs* was significantly upregulated in both qPCR and TMT analysis. Between 3dwb and 12dwb, *sspA* was significantly downregulated in both qPCR and TMT analysis, indicating a relative consistency with the TMT data for these proteins. However, for genes (*SA0914, femX* and *sdhB*) there was a dysregulation between qPCR and TMT data (Table S7).

## 3. Discussion

*S. aureus* biofilm development on medical devices [3,4,5,33], healthcare equipment [34,35] and surfaces [34,36,37,38,39,40] is common but potential molecular markers and mechanisms associated with the formation of *S. aureus* biofilm are still poorly understood. Significant variations in the expression of the proteomic profile of *S. aureus* relating to numerous metabolic and cellular processes, virulence factors, and transporter factors have been reported for young *S. aureus* biofilm compared to planktonic growth mode [19,21,41] but little work has been done comparing *S. aureus* biofilm of different ages. In this TMT-based high-resolution proteomics study, we mainly focused on the profiles of the protein expression in the 3-day biofilm and 12-day biofilm of *S. aureus* as compared to the planktonic growth mode.

Further, we also performed qPCR experiments on some selected significantly dysregulated genes to explore the correlation between qPCR and TMT data. Gene expression levels utilising qPCR were found to be comparably consistent with the data obtained from TMT-based MS analysis for some of the proteins but not for all proteins. The lack of an association between transcriptomics and proteomics is likely attributable to differences in half-lives and post-transcription machinery. Moreover, the absence of any correlation between data obtained from TMT and qPCR analysis could be due to differences in cell lysis and extraction methods performed on the samples.

**Figure 5:**
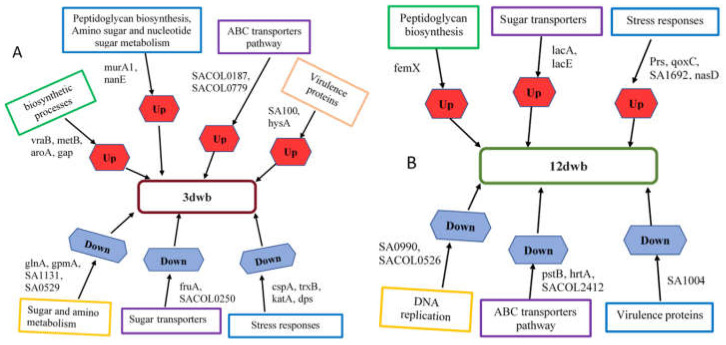
Novel regulators of *S. aureus* biofilm formation in (**A**) 3-day biofilm, and (**B**) 12-day biofilm identified from TMT-based analysis.

Proteomic profiling analysis at various phases of biofilms highlighted interesting findings on metabolic processes, biosynthetic processes, transport systems such as sugar and ABC transporters, and stress response (e.g., *gap*, *nanE*, *nasD*, *SA1692*) involved in *S. aureus* biofilm formation (Figure 5).

**Figure 6:**
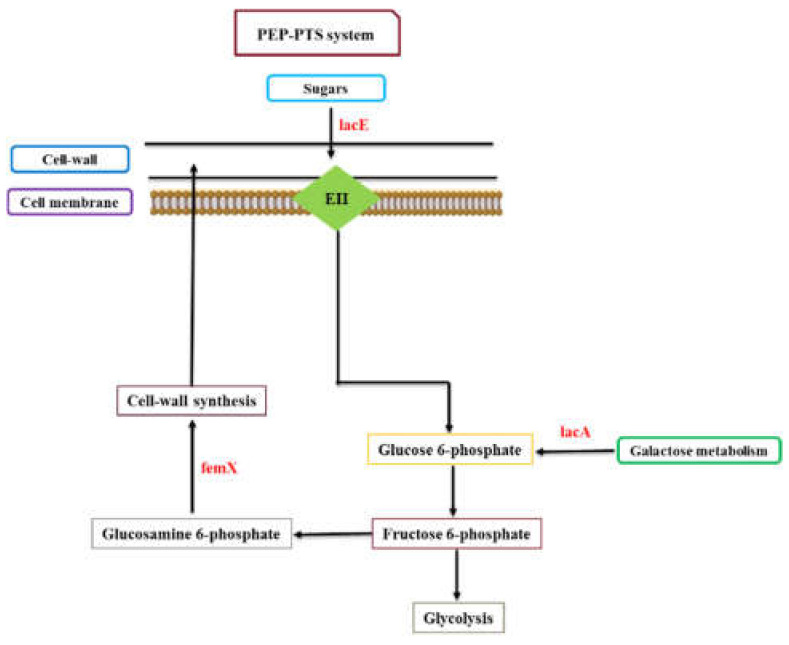
Linking pathway demonstration of phosphotransferase (PTS), galactose metabolism, and peptidoglycan biosynthesis in 12dwb. Bacterial biofilm can uptake sugars by specific sugar transport systems (e.g., PTS) and non-PTS systems (e.g., galactose metabolism). *LacAE* is involved in the metabolism of sugar molecules via fructose 6-phosphate which is also linked with glycolysis and synthesis of cell-wall components. In addition, peptidoglycan biosynthesis proteins (e.g., *femX*) are also directly linked with cell-wall formation. EII: Enzyme II; Red names denote encoded genes with increased abundance in 12dwb.

For instance, the upregulation of 3-phosphoshikimate 1-carboxyvinyltransferase (*aroA*) involved in the shikimate pathway shows an enhanced synthesis of aromatic amino acids in 3dwb. Some studies demonstrated that *aroA* production can result in glyphosphate resistance in pathogenic bacteria such as *S. aureus* and the plant pathogen *Burkholderia glumae’s* growth, virulence, and UV tolerance [42,43,44]. This suggests that a higher accumulation of glyphosphate in biofilm may show increased resistance levels to glyphosphate and virulence mechanisms, probably resulting in enhanced biofilm formation. PIA is a major component of the biofilm matrix, and its formation also depends on the phosphotransferase system (PTS) a distinct method used by bacteria to uptake sugar. The phosphoenolpyruvate-protein (PEP) PTS is a carbohydrate-specific active transport mechanism and is responsible for transporting sugar substrates (such as glucose, lactose, fructose, and cellobiose) during translocation across the cell membrane. We observed interesting findings with sugar transporter systems such as PTS, upregulation of lactose-specific transporter subunits IICB (*lacE*), galactose-6-phosphate isomerase (*lacA*) involved in galactose metabolism (Figure 6). Inhibition of PTS systems has been shown to reduce the virulence of *S. aureus* in a murine model [45]. Aminoacyltransferase (*femX*) is involved in peptidoglycan biosynthesis, by incorporating L-amino acids into the interchain cross-bridge of peptidoglycan chains, thus stabilizing the peptidoglycan [46] and was upregulated in the 12-day old biofilm (Figure 6).

*FemX* incorporates L-amino acids into the interchain cross-bridge of peptidoglycan chains, thus stabilizing the peptidoglycan Gram-positive organisms such as *S. aureus* [47,48]. *Fem* family enzymes are essential for the expression of β-lactam resistance mediated by low-affinity penicillin-binding protein family (PBPs) [48], and upregulation of *FemX* could be one possible mechanism by which more mature biofilm increase cell-wall protein formation resulting in increased EPS deposition, which increases tolerance to antimicrobials.

ABC transporter system proteins such as peptide ABC transporter substrate-binding protein encoded by SACOL0187 and heme ABC transporter ATP-binding protein (*SACOL0779*) were significantly upregulated in 3dwb, whereas hemin ABC transporter ATP-binding protein (*hrtA*) and amino acid ABC transporter substrate-binding protein (SACOL2412) are significantly downregulated in 12dwb. ABC transporter proteins play important roles in the delivery of molecules required for the maintenance of cellular nutrient supply and integrity. It is likely that, during early biofilm development, the bacteria are more metabolically active and biofilm formation requires a better nutrient supply, thus the ABC transporter proteins are upregulated. On the other hand, as biofilm ages and matures, the cells are less metabolically active and so fewer nutrients are required. Numerous proteomics and transcriptomics studies have reported the differential expression of ABC transporter proteins during biofilm development in various bacteria including *S. aureus* [20,49,50,51,52].

In addition, several ABC transporter proteins have been shown to play a role in *S. aureus* pathogenesis and antimicrobial resistance [53,54,55,56,57]. An ABC transporter acts as a positive regulator during biofilm development in acute and chronic *S. aureus*-associated osteomyelitis in the pig [57], whilst it is a negative regulator of biofilm formation for *Listeria monocytogenes* [50].

Similarly, KEGG pathway analysis indicated a higher metabolic activity profile in 3dwb compared to 12dwb, with small molecules, amino sugar and nucleotide metabolism, amino acid biosynthesis and metabolism, and energy metabolism associated proteins being significantly upregulated, in 3dwb compared to 12dwb. While translation, post-translational modification, ribosomal, cell division, attachment, and cofactor metabolic process associated proteins were significantly downregulated, indicating a lower metabolic activity profile in 12dwb. This also suggested that the more mature biofilm is more adapted to adherent growth and utilized alternative metabolic pathways during biofilm development. The metabolically active biofilm growth mode that we observed in 3dwb has less mass and is easier to disperse than 12dwb. This is supported by Suriyanarayanan et al. (2018) who compared the proteome of weak biofilm producers versus strong biofilm producers from *Enterococcus faecalis* [44]. They reported that strong biofilm producers showed lower metabolic activity compared to weak biofilm producers and suggested that it may be likely that the metabolically active biofilm cells (weak biofilm producer) result in less mass and promote dispersal rather than adhesion.

Biofilms are organized as tightly packed communities with bacteria exhibiting different metabolic states partly due to the availability of nutrients and oxygen declines gradually due to consumption and impairment of diffusion and is thus limited in the biofilm’s deeper layers [18]. It was, therefore, not surprising that we found nitrite reductase encoded by *nasD* significantly upregulated in 12-day biofilm which is responsible for the production of energy under oxygen-limited environments. [18,41,52,58,59,60].

The expression of PIA synthesizing proteins *IcaADBC* is influenced by oxygen availability and nutrient, cell density, and numerous stress responses, and we observed several stress responses associated proteins were differentially regulated in 3-day biofilm (such as SA0558, helicase *DnaB*, aldo/keto reductase, SACOL0959, response regulator *GraR*, *trxB*, glyoxalase, *katA*, *dps*, etc.), and in 12-day biofilm (such as *SACOL1561*, *qoxC*, *SA1692*, *SA0758*, glyoxal reductase, etc.). We observed that oxidative stress-associated proteins were more downregulated in 3-day biofilm; this has also been shown by Graf et al., 2019 [18]. In addition, due to oxygen limitation and oxidative stress within the biofilm, cytochrome C oxidase subunit III (*qoxC*), was significantly upregulated in the 12-day biofilm, and is involved in electron transport chain maintenance. A recent proteomic study by Lei et al., (2017), emphasizes the importance of stress response-associated proteins for virulence regulation, and adaptation during chronic infection in a rat model of orthopedic implant-associated biofilm infection from *S. aureus* [61].

A limitation of this study is that we only compared 3-day and 12-day biofilms. Future studies can include even more prolonged biofilms, such as one-month, 3-month and 6-month biofilms to see the trend of proteome changes with aging. Another limitation of this study is that only one strain of *S. aureus* was used. However, we chose an ATCC reference strain that is commonly used for standard biocide testing and permits other laboratories to replicate our work. In this initial work, we have determined differentially expressed proteins using this reference strain; therefore, future work could compare these protein concentrations using different platforms, capable of comparing multiple *S. aureus* strains, such as targeted ELISAs or biochemical assays.

Numerous exogeneous virulence factors such as: coagulase, hyaluronate lyase, staphostatin A, catalase, phenol soluble modulin, alpha-hemolysin, staphyloxanthin, serine protease, aureolysin, thermonuclease, and lipase were upregulated in 3dwb compared to 12dwb. Quantitative analysis of these virulence factors can also be done in future studies to validate the proteomics results in multiple *S. aureus* strains.

## 4. Materials and Methods

The objective of this research was to create quantitative proteomic profiling to define the distinctions between *S. aureus* cells that progress through the lifestyles of planktonic and biofilm. To accomplish this, we conducted protein extraction and fractionation, reduction, alkylation and in-solution digestion that enabled us to produce extremely complicated samples that were analyzed with TMT-based mass spectrometry. Three biological replicates were analyzed for each growth condition: planktonic, mature and prolonged biofilms.

### 4.1. Microorganism and Culture Conditions

*Staphylococcus aureus* reference strain (ATCC 25923) was grown to stationary phase in 100% tryptic soy broth (TSB) for 24 h with constant agitation at 130 rpm and 37 °C. *S. aureus* biofilm was grown on removable polycarbonate coupons in the Centers for Disease Control (CDC) biofilm reactor (BioSurface Technologies Corp, Bozeman, MT, USA), in batch phase, at 37 °C, initially with 50% TSB for 48 h. At 48 h the media was drained and replaced with 20% TSB which was repeated every 48 h as necessary to produce 3-day and 12-day biofilms. Shear was provided by baffle rotation at 130 rpm. Biofilm was grown and harvested from three separate experiments.

### 4.2. Protein Extraction and Fractionation

Protein extraction and fractionation were modified from a previously described method [62]. Planktonic bacteria were pooled from three independent growth of 24 h *S. aureus* cultures and were mixed with lysis buffer containing 100 mM triethylammonium bicarbonate (TEAB; Sigma-Aldrich, St. Louis, Missouri, MO, USA) pH 8.5 and 1% (*w/v*) sodium deoxycholate (Sigma-Aldrich) at 10:1 ratio (supernatant: lysis buffer), while *S. aureus* biofilm coated coupons (*n* = 24) were washed to remove non-adherent cells and coupons individually placed in 2 mL of phosphate-buffered saline (PBS) and lysis buffer and incubated overnight with gentle shaking at 4 °C. Samples were probe sonicated in an ice-cold environment (Sonic Ruptor, Omni International, Kennesaw, Georgia, GA, USA) for 2 min at 50% power and 70% pulses. The samples were centrifuged at 12,000× *g* for 10 min and the supernatant was filtered through a 10 kDa molecular weight cut off (MWCO) ultra-membrane filter tube (Sigma-Aldrich) before centrifugation at 4000× *g* for 20 min. Protein samples were washed three times with PBS to remove TSB and lysis buffer and further concentrated using a 3 KDa MWCO filter tube (Sigma Aldrich).

The BCA protein assay (Thermo Fisher Scientific, Waltham, MA, USA) was used to measure protein concentration at 562 nm wavelength as per the manufacturer’s instructions.

### 4.3. Protein Reduction, Alkylation, and Digestion

A total of 40 µg protein of each biological replicate was reduced with 5 mM DTT for 15 min at room temperature and alkylated with 10 mM iodoacetamide (IAA) in the dark for 30 min at room temperature. Alkylated samples were diluted with 100 mM TEAB pH 8.5. Samples were digested overnight at room temperature by the addition of Lys-C at a ratio of 1:30 followed by the addition of trypsin at a ratio of 1:30 for 5.5 h at 37 °C. Samples were adjusted to 1% (*v*/*v*) TFA, and the precipitated deoxycholate was removed by centrifugation. Samples were centrifuged at 14,100× *g* and desalted with 0.2% TFA washing using 3M-Empore SDB-RPS Stage Tips (Thermo Fisher Scientific). Samples were eluted with 5% ammonium hydroxide in 80% acetone and centrifuged at 1000× *g* for 5 min, vacuum dried and stored at −20 °C until further processing.

### 4.4. TMT Labeling and High pH Fractionation

Tandem Mass Tag (TMT; Thermo Fisher Scientific) reagents (0.8 mg) were dissolved in acetone (85 µL) of which 41 µL was added to the reconstituted (100 µL of 100 mM TEAB pH 8.5) samples and incubated for 1 h at RT. A volume of 8 µL of 5% hydroxylamine was added to each TMT-labelled sample and incubated for 15 min at RT. A volume of 2 µL of each labelled sample was pooled, vacuum-dried, and reconstituted in 30 µL 0.1% formic acid (Merck, Kenilworth, NJ, USA) solution, centrifuged for 5 min at 14,000× *g* and analysed with a mass spectrometer (for detailed information see nanoLC-ESI-MS/MS using Orbitrap Elite).

Data searching was conducted using Proteome Discoverer 1.3 (Thermo Fisher Scientific, for detailed information, see data processing). Based on the applied normalization values from this search result an equal number of peptides were taken from each sample, pooled and vacuum dried (miVac). The dried, labelled sample was resuspended in buffer A (5 mM ammonia, pH 10.5) and fractionated by high pH reverse phase-high pressure liquid chromatography (RP-HPLC; Agilent Technologies, Santa Clara, CA, USA). The dried, labelled sample was resuspended in buffer A. After sample loading and washing with 97% buffer A for 10 min, the concentration of buffer B (5 mM ammonia solution with 90% Acetone, pH 10.5) was increased from 3% to 30% for 55 min; 70% for 10 min; 90% for 5 min at a flow rate of 300 µL/min. The eluent was collected every 2 min at the beginning until 16 min and every 1 min intervals for the remainder of the gradient. The fractionated sample was pooled into 19 fractions and dried in miVac. Finally, each fraction was resuspended in 55 µL of 0.1% FA for MS analysis.

### 4.5. Nanoflow LC-ESI-MS/MS

All samples were run on two sequential mass spectrometer systems Orbitrap Elite (Thermo Fisher Scientific) and Q Exactive (Thermo Fisher Scientific).

#### 4.5.1. Nanoflow LC-ESI-MS/MS Using Orbitrap Elite

An Orbitrap Elite (Thermo Fisher Scientific) mass spectrometer equipped with PicoView 550 Nanospray Source (New Objectives, Littleton, MA, USA), an Eksigent ultra-pressure liquid chromatography (UPLC) system (AB SCIEX, Framingham, MA, USA) consisting of an ekspert™ nanoLC 425 UPLC pump and ekspert™ nanoLC 400 (Thermo Fisher Scientific) autosampler was used for acquiring data. 20 µL of each fraction was loaded onto a self-packed 100 µm × 3.5 cm trap column with Halo^®^ 2.7 µm 160 Å ES-C18 (Advanced Materials Technology, Wilmington, DE, USA) and desalted with loading buffer [0.1% FA] at a flow rate of 4 µL/min for 10 min. Peptides were eluted onto a self-packed analytical column 100 µm × 30 cm with Halo^®^ 2.7 µm 160 Å ES-C18 (Advanced Materials Technology) with the linear gradients of mobile phase A (0.1% FA/5% DMSO) and mobile phase B (0.1% FA/5% DMSO) starting with B (1–10%) for 0.1 min, B (10–20%) for 52 min, B (20–32%) for 48 min followed by (32–43%) for 20 min with a flow rate of 450 nL/min across the gradient. The eluent from the trap was diluted with 100 nL/min of buffer A before reaching the analytical column. The peptides refocused and separated over the analytical column at 60 °C. The peptides were ionized by electrospray ionization, and data-dependent MS/MS acquisition was carried out using an Orbitrap Elite (Thermo Fisher Scientific) consisting of 1 full MS1 (R = 120 K) scan acquisition from 380 to 1600 *m*/*z*, and 15 higher-energy collisional dissociations (HCD) type MS2 scans (R = 30 K).

#### 4.5.2. Nanoflow LC-ESI-MS/MS Using Q Exactive

A Q Exactive (Thermo Fisher Scientific) Mass Spectrometer equipped with Nano spray Source and Easy nLC 1000 (Thermo Fisher Scientific) was used for acquiring data. 10 µL of each fraction was loaded onto a self-packed 100 µm × 3.5 cm reversed-phase peptide trap with Halo^®^ 2.7 µm 160 Å ES-C18 (Advanced Materials Technology) desalted with 20 µL of loading buffer (0.1% FA) and the peptide trap was then switched on-line with the analytical column a self-packed 75 µm × 3.5 cm Halo^®^ 2.7 µm 160 Å ES-C18 column. Peptides were eluted with the linear gradients of mobile phase A (0.1% FA) and buffer B [100% (*v*/*v*) Acetone, 0.1% (*v*/*v*) FA] starting with (1–30%) for 110 min, B (30–85%) for 2 min followed by 85% B for 8 min with a flow rate of 300 nl/min. Peptides were ionized by electrospray ionization and data-dependent MS/MS acquisition was carried out using a Q-Exactive consisting of 1 full MS1 (R = 70 K) scan acquisition from 350 to 1850 *m*/*z*, and 10 HCD type MS2 scans (R = 70 K).

### 4.6. Database Search, Statistical Analysis, and Bioinformatics

The raw data files were submitted to Proteome Discoverer (version 1.3, Thermo Fisher Scientific). The data were processed using Sequest and Mascot (Matrix Science, London, UK) against *S. aureus* reference strain (ATCC 25923) from Genbank CP009361 and CP009362. For protein identification, the following options were used: peptide mass tolerance = 10 ppm; MS/MS tolerance = 0.1 Da; enzyme = trypsin, missed cleavage = 1; fixed modification, carbamidomethyl (C), TMT10-plex (K) and TMT10-plex (N-term); variable modification, oxidation (M), Deamidated (N, Q) and Acetyl (N-Terminus). Quantification was performed based on the peak intensities of reporter ions in the MS/MS spectra. Below 1% false discovery rate was selected as the cut-off for peptide identification. Protein quantification was based on the total intensity of the assigned peptides. After the extraction of protein ratios with Proteome Discoverer, additional processing and statistical analysis were done using the TMTPrePro R package [2]. BLAST search was performed using highly annotated strains *S. aureus* N315 and *S. aureus* COL. Proteins were considered upregulated when the TMT ratio was above 1.5 and downregulated when the TMT ratio was lower than 0.66 in biofilm growth compared to planktonic growth with significant *p*-Value < 0.05. Significant differentially expressed proteins (>2-fold) were determined by using VENNY (v.2.1) (http://bioinfogp.cnb.csic.es/tools/venny/, accessed on 25 June 2018) and processed further to gain more functional insights. Metabolic pathways of identified proteins were analysed by using Kyoto Encyclopedia of Genes and Genomes (KEGG) mapper (https://www.genome.jp/kegg/tool/map_pathway2.html, accessed on 6 August 2018). Subcellular localisation of identified proteins was analysed by using PSORTb (version 3.0.2) (http://www.psort.org/psortb/index.html, accessed on 23 January 2018) [3]. The protein–protein interaction (PPI) network of significantly differentially expressed proteins was analysed by STRING software v.10.0 (http://string-db.org/, accessed on 25 June 2018) [4].

### 4.7. Validation of TMT Data with qPCR Results

Five genes encoding chitinase *SA0914*, glutamyl endopeptidase *sspA*, ribose-phosphate pyrophosphokinase *prs*, aminoacyltransferase *femX*, and succinate dehydrogenase *sdhB* were chosen as targets to analyse the levels of RNA expression to validate the expression differences in planktonic and biofilms. The 16S rRNA was used as an endogenous control to normalize the data and the level of differential expression of the five genes between planktonic and biofilm was compared.

RNA was extracted from *S. aureus* planktonic, 3dwb, and 12dwb using an RNeasy Mini Kit (Qiagen, Hilden, Germany) with RNAlater to prevent degradation and RNase-free DNase treatment to remove the genomic DNA. RNA concentration was determined by absorbance at 260 nm, and quality was assessed by absorbance ratio (A260/A280). A total of 200 ng of RNA was used for cDNA synthesis using the SuperScript™ IV VILO™ Master Mix (Thermo Fisher Scientific). Real-time (RT)-PCR primers used in this study (Table S8) were designed following *S. aureus* (ATCC 25923) genome sequence Genbank accession number CP009361 and CP009362.

Quantitative real-time PCR (qPCR) was conducted in an Applied Biosystems quantitative real-time PCR machine (ViiA™ 7 qPCR, Thermo Fisher Scientific) in duplicate on two biological replicates. A volume of 25 μL of qPCR reaction mix containing 12.5 μL 2X PowerUp™ SYBR™ Green Master Mix (Thermo Fisher Scientific), 1 μL each of 10 μM reverse and forward primer for a final primer concentration of 400 nM, 8.5 μL of water and 2 μL of 1:5 diluted cDNA. Controls in each run included a no-template control (NTC) for each primer set, which consisted of all PCR components except cDNA template which was replaced by nuclease-free water. A no reverse transcription control (no RT control) was also included in initial experiments for each targeted gene. No RT controls consisted of the cDNA sample with no reverse transcriptase enzyme to determine if there was contaminating genomic DNA in the RNA. Cycling conditions for real-time PCR were set as an initial activation step of 95 °C for 10 min to activate the polymerase, followed by 40 cycles of denaturation at 95 °C for 15 s, annealing at 50 °C or 55 °C for 40 s and extension at 72 °C for 30 s, or annealing and extension at 60 °C for 1 min.

The expressed copy number of each target gene was normalized to 16s rRNA copy number within the same growth condition first, and the levels of candidate gene expression of planktonic and biofilms (3dwb and 12dwb) were compared to study relative gene expression using a previously described method [63]. The ratios from the qPCR results were obtained by comparing with planktonic in 3dwb and 12dwb.

## 5. Conclusions

In this study, the comparison of stationary phase planktonic bacteria with 3-day biofilm and 12-day biofilm demonstrated a significant range of quantitative proteomic shifts. Significantly upregulated proteins in the 3-day biofilm suggested a higher metabolic activity while proteins linked with energy metabolism (*PTS*, *lacAE*), and cell-wall synthesis (*femX*) were upregulated in the 12-day biofilm and may be one possible mechanism by which more mature biofilm increases resistance to antimicrobials.

Our analyses provide a new understanding of the impact on the protein expression profile of growth mode for both planktonic and different stages of biofilm, potential biofilm regulators and the probable mechanisms for the development of *S. aureus* biofilms.

## Data Availability

Data are available via ProteomeXchange [64] with identifier PXD033499.

## Supplementary Materials

The following supporting information can be downloaded at: https://www.mdpi.com/article/10.3390/ijms23126415/s1.
