# Supplementary material for: Proteome of Staphylococcus aureus Biofilm Changes Significantly with Aging
Source: Int J Mol Sci. 2022 Jun 8;23(12):6415. doi: 10.3390/ijms23126415 (PMC9223533; doi:10.3390/ijms23126415)
Supplement: Supplementary file 1 [file ijms-1708554-supplementary Figure S1.pdf › ijms-1708554-supplementary Figure S1.pdf]

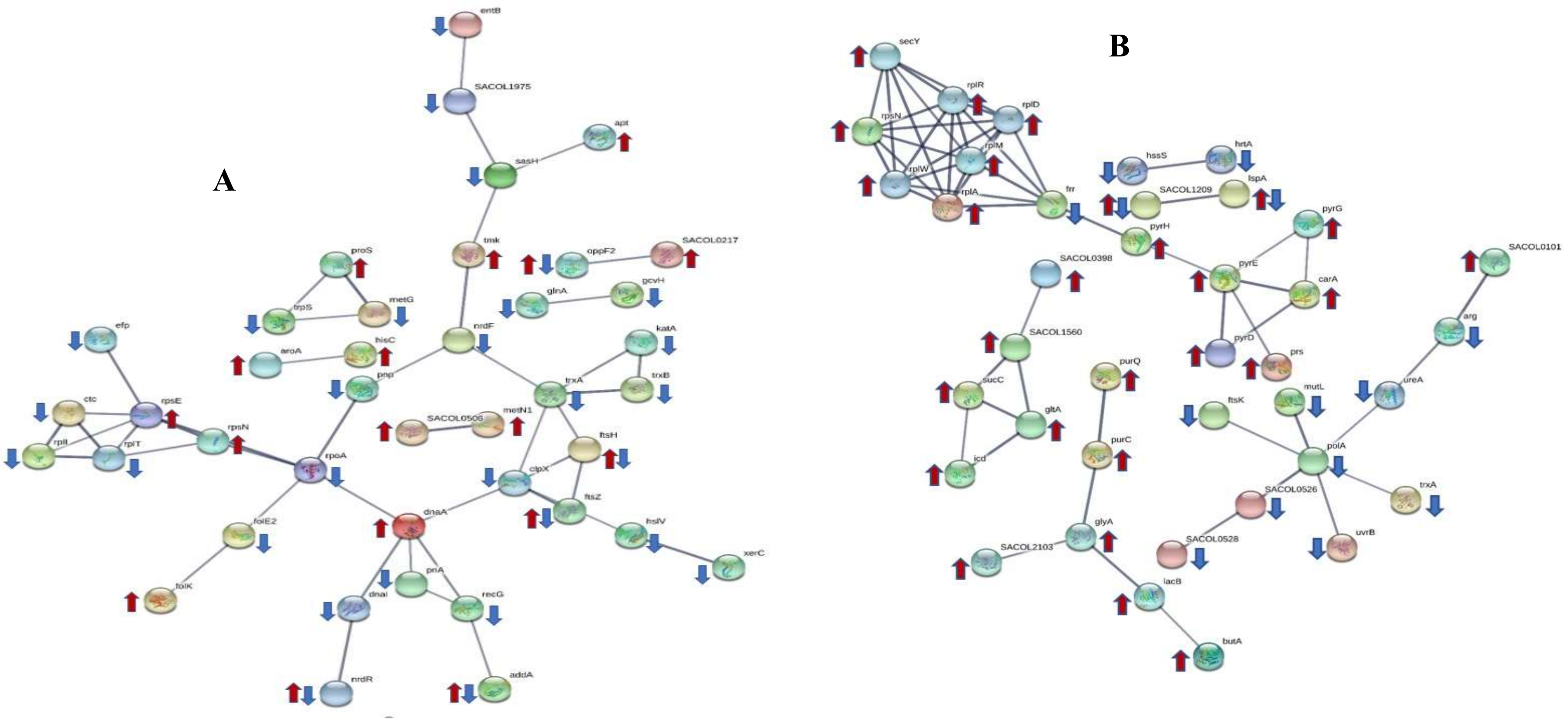

Figure S1: The protein-protein interaction network as analyzed by STRING software with high confidence (0.700) and omitted nodes those are not connected to the network. Red arrows represent upregulation and blue arrows represent downregulation. The map of the exclusively upregulated and downregulated proteins from (A) 3dwb and (B) 12dwb show high interactions, which indicates that the proteins are biologically linked, at least partly, as a group.
